# Synthesis of patient-specific multipoint 4D flow MRI data of turbulent aortic flow downstream of stenotic valves

**DOI:** 10.1038/s41598-022-20121-x

**Published:** 2022-09-26

**Authors:** Pietro Dirix, Stefano Buoso, Eva S. Peper, Sebastian Kozerke

**Affiliations:** Institute for Biomedical Engineering, University and ETH Zurich, Zurich, Switzerland

**Keywords:** Biomedical engineering, Fluid dynamics

## Abstract

We propose to synthesize patient-specific 4D flow MRI datasets of turbulent flow paired with ground truth flow data to support training of inference methods. Turbulent blood flow is computed based on the Navier–Stokes equations with moving domains using realistic boundary conditions for aortic shapes, wall displacements and inlet velocities obtained from patient data. From the simulated flow, synthetic multipoint 4D flow MRI data is generated with user-defined spatiotemporal resolutions and reconstructed with a Bayesian approach to compute time-varying velocity and turbulence maps. For MRI data synthesis, a fixed hypothetical scan time budget is assumed and accordingly, changes to spatial resolution and time averaging result in corresponding scaling of signal-to-noise ratios (SNR). In this work, we focused on aortic stenotic flow and quantification of turbulent kinetic energy (TKE). Our results show that for spatial resolutions of 1.5 and 2.5 mm and time averaging of 5 ms as encountered in 4D flow MRI in practice, peak total turbulent kinetic energy downstream of a 50, 75 and 90% stenosis is overestimated by as much as 23, 15 and 14% (1.5 mm) and 38, 24 and 23% (2.5 mm), demonstrating the importance of paired ground truth and 4D flow MRI data for assessing accuracy and precision of turbulent flow inference using 4D flow MRI exams.

## Introduction

Aortic stenosis (AS) is a common condition associated with high morbidity and mortality^1,2^. Early detection and treatment of AS are associated with lower mortality rates, but the correct classification of the disease severity remains a challenge^2^. Since cardiovascular pathologies are usually associated with abnormal flow patterns^3–5^ and irreversible pressure losses^6–10^, the analysis of aortic flow fields is considered an important element for risk stratification and personalized planning of clinical interventions.

Cardiovascular magnetic resonance (CMR), and in particular phase-contrast (PC) MRI, has enabled measurements of time-resolved volumetric flow patterns (4D flow MRI)^11^ in research and clinical settings. Despite recent advancements in sequence design^12–14^ and image reconstruction methods^15^, data is limited by spatiotemporal resolution and artifacts. Therefore, the development of robust and realistic models for the analysis of 4D flow MRI datasets is a fundamental step to enable predicting accuracy and precision of such measurements in research and clinical routine.

Deep learning (DL) methods are particularly suitable to discover intricate patterns in large datasets^16,17^, which makes them ideal candidates to infer flow parameters and patterns contained in highly dimensional and complex 4D flow MRI exams. Recent works on image reconstruction^15^, segmentation^18,19^, classification^20^ and flow super-resolution^21^ have demonstrated the potential of DL algorithms. Berhane et al.^18^ and Bratt et al.^19^ used fully automated segmentation algorithms trained on manually labeled cine 2D and 4D flow MRI datasets to accelerate flow and diameter measurements in the aorta. However, the scarcity of high-quality labeled training datasets^22^ effectively hampers the implementation of DL based inference approaches for 4D flow MRI. Fries et al.^20^ alleviated the burden of obtaining manually labeled datasets by developing a weakly supervised DL model for classification of aortic valve malformations based on a small number of manually annotated scans. Other works have demonstrated the viability of augmenting clinical datasets using synthetic images^23,24^ as the training of inference machines is significantly compromised by the limited number and potentially biased distributions of paired ground truth and imaging data. In general, however, the incorporation of manually labeled datasets as well as inherent uncertainties in the MRI measurements lead to biased and imperfect “ground truth” data. This suggests that the intrinsic accuracy and precision of methods developed using such training datasets cannot be assessed and only approximate metrics can be derived using in-situ and in-vitro experiments^5^.

Fluid flow can be obtained by simulating hemodynamics in realistic aortic shapes^25–27^. The corresponding MR signal is derived by simulating the acquisition process using the simulated data as input, effectively creating trustworthy ground truth and MR image pairs^21,28,29^. In Ferdian et al.^21^, downsampled synthetic flow fields were derived from data generated by computational fluid dynamics (CFD) and used to train a super-resolution algorithm capable of estimating high-resolution flow features from low-resolution data. Inference was limited to velocity fields and turbulence was not incorporated. A more realistic approach consists of using data from CFD to compute the trajectories of individual material points while their complex-valued magnetization, and corresponding MRI signal, can be evaluated by solving the Bloch equations in the Lagrangian frame of reference^30,31^. Such a method can be used to evaluate specific MRI sequences while inherently accounting for flow-induced displacement and dephasing artifacts^32^. However, in order to accurately estimate MRI signals for turbulent flows, a large number of material points need to be tracked, rendering these simulations computationally expensive. Alternatively, synthetic MRI images can be obtained using a model equation for the signal, which directly includes pointwise velocity and turbulence data from CFD, drastically reducing the computational cost^33,34^.

The presence of transitional or turbulent flow regimes downstream of aortic stenoses^5^ suggests that simulations including turbulence modeling are an important step towards accurate modeling of pathological aortic flows. However, to the best of our knowledge, synthesis of 4D flow MRI data using turbulent flow simulations in realistic moving aortic shapes and signal encoding of velocity magnitude, phase and intra-voxel standard deviation (IVSD) has not been performed so far.

In this work we propose a framework to synthesize 4D flow MRI datasets of turbulent flow in the aorta with moving walls. Ground truth velocity and turbulence fields, computed with CFD, are input to generate multipoint MR signals at realistic resolution followed by Bayesian image reconstruction to output velocity and turbulence maps. The method is utilized with steady and pulsatile flow in idealized stenotic geometries to investigate the impact of the interplay of signal-to-noise ratio (SNR), spatial resolution and time averaging on measurement accuracy and precision of turbulent kinetic energy (TKE). Successively, patient-specific aortic 4D flow MRI data is generated with various degrees of aortic stenosis to report errors relative to ground truth for realistic SNR and resolutions.

## Results

### Synthetic 4D flow MRI data generation

**Figure 1:**
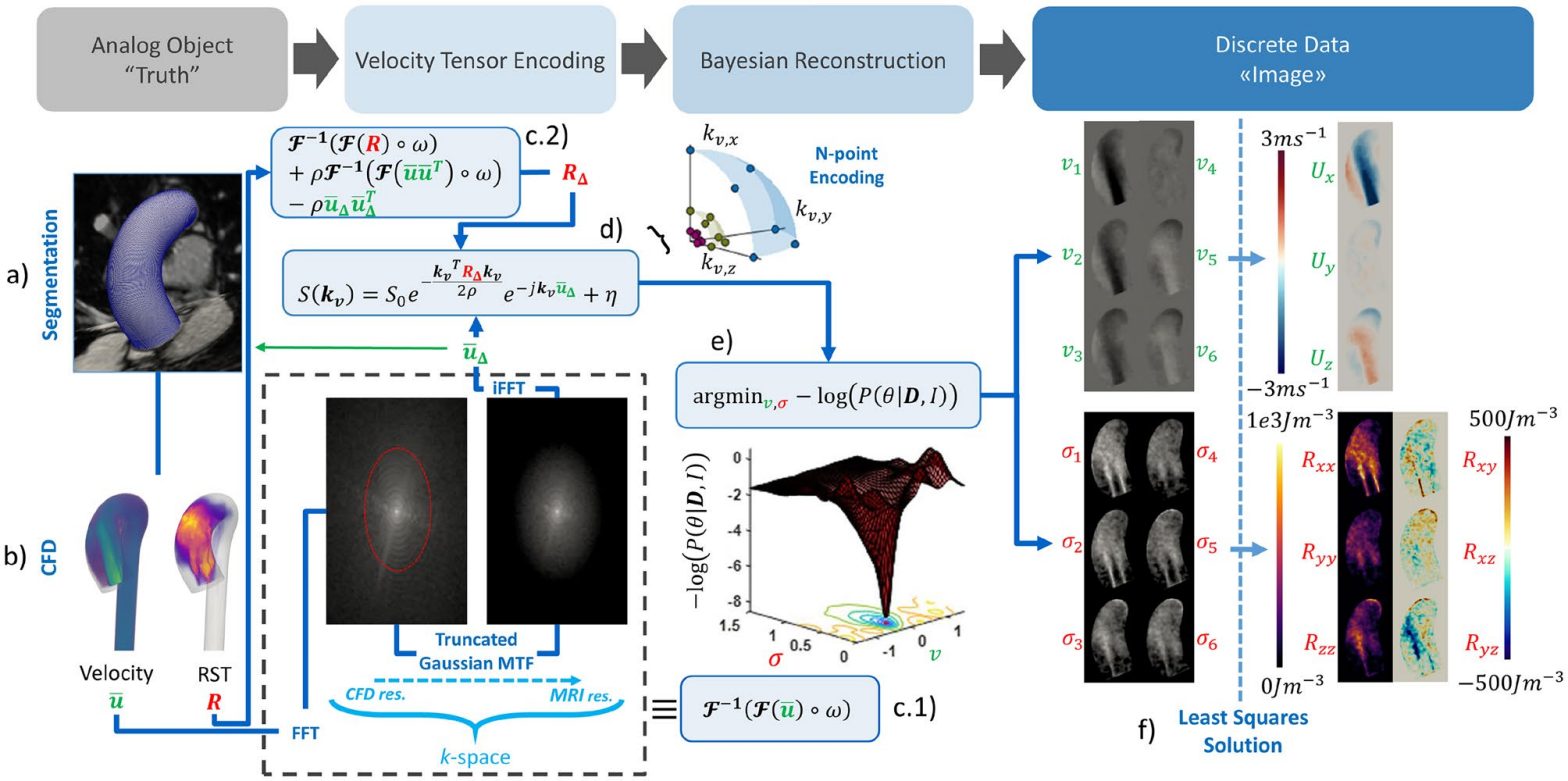
Pipeline for generation of synthetic patient-specific pulsatile mean and turbulent 4D flow MRI datasets. (**a**) Patient-specific segmentation and mesh generation. (**b**) Large Eddy Simulation CFD simulation to obtain mean velocity $\overline{u }$ and Reynolds shear tensor $R$. (**c**) Band-limited projection of velocity (${\overline{u} }_{\Delta }$) and Reynolds stress tensor (${R}_{\Delta }$) in the spatial frequency domain (k-space) upon Fourier transform $\mathcal{F}$. (**d**) Signal model to generate MRI signal, S, for a given velocity encoding vector, ${{\varvec{k}}}_{{\varvec{v}}}$, fluid density, $\rho$, and complex-valued white Gaussian noise, $\eta$. (**e**) Bayesian reconstruction of voxel mean velocities, $\nu$, and intra-voxel variances and co-variances, ${\sigma }^{2},$ and (**f**) their projection onto Cartesian coordinates using a least squares solution approach to obtain mean velocity vector $U$ and Reynolds stress tensor $R$.

Figure 1 illustrates the overall pipeline for the generation of synthetic 4D flow MRI data. 2D cine MRI and time-resolved 2D PC-MRI data are utilized to extract transient moving aortic geometries and corresponding inlet velocity profiles (Fig. 1a). A large eddy simulation (LES) CFD approach with moving boundaries is employed to simulate turbulent flow (Fig. 1b). Subsequently, multipoint MRI signals are synthesized using dedicated signal models (Fig. 1c,d) followed by reconstruction using a Bayesian approach (Fig. 1e). Finally, velocity and intra-voxel standard deviation data are projected onto Cartesian coordinates to output velocity, Reynolds stress tensor (RST) and TKE maps (Fig. 1f). In this work both idealized and realistic shapes are used. The former allow to define control cases to study the effect of the interplay of SNR, resolution and time averaging for a given scan time budget, while the latter exemplify the utility of the method for patient-specific studies.

### Spatiotemporal resolution and SNR dependencies in steady stenotic flow

**Figure 2:**
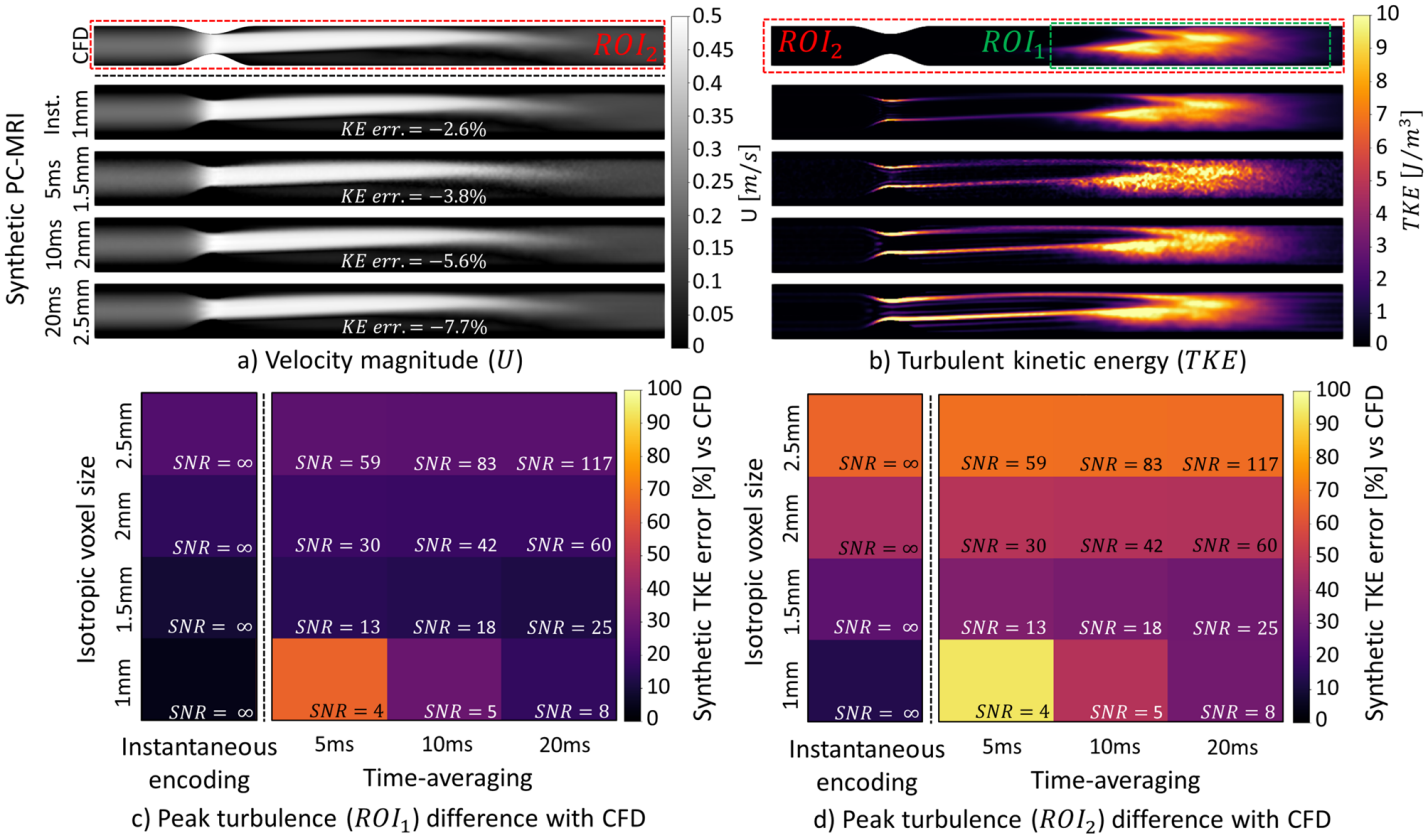
Impact of SNR and spatiotemporal resolution on velocity and turbulent kinetic energy for a 75% eccentric stenosis and steady flow. Magnitude of velocity $\left(U\right)$ (**a**) and turbulent kinetic energy $\left(TKE\right)$ (**b**) for varying voxel sizes, time averaging and, correspondingly, signal-to-noise ratios (SNR) are shown. The percentage error in total TKE as a function of spatial resolution, time averaging, and SNR is shown in (**c**) and (**d**) for $RO{I}_{1}$ (envelope of the turbulent region) and $RO{I}_{2}$ (whole geometry), respectively. Instantaneous (Inst.) encoding refers to a hypothetical noise-free PC-MRI experiment with infinitely high velocity encoding bandwidth.

In Fig. 2, the effect of spatial resolution and time averaging on velocity and TKE quantification for steady flow is visualized. Of note, a fixed hypothetical scan time budget is assumed in all MRI synthesis experiments and hence $\mathrm{SNR}\propto V\sqrt{\Delta t}$, where $V$ denotes voxel volume and $\Delta t$ temporal averaging. For isotropic voxel sizes between 1 and 2.5 mm and hypothetical instantaneous encoding, total kinetic energy (KE) is underestimated by up to 8% while total TKE is overestimated by up to 24% in ${\mathrm{ROI}}_{1}$ (envelope of the turbulent region, Fig. 2c) and by 13–65% for ${\mathrm{ROI}}_{2}$ (whole geometry, Fig. 2d). For SNR values between 30 and 4, noise contribution to total TKE varies from 14 to 94% for ${\mathrm{ROI}}_{1}$.

### Spatiotemporal resolution and SNR dependencies in pulsatile stenotic flow

**Figure 3:**
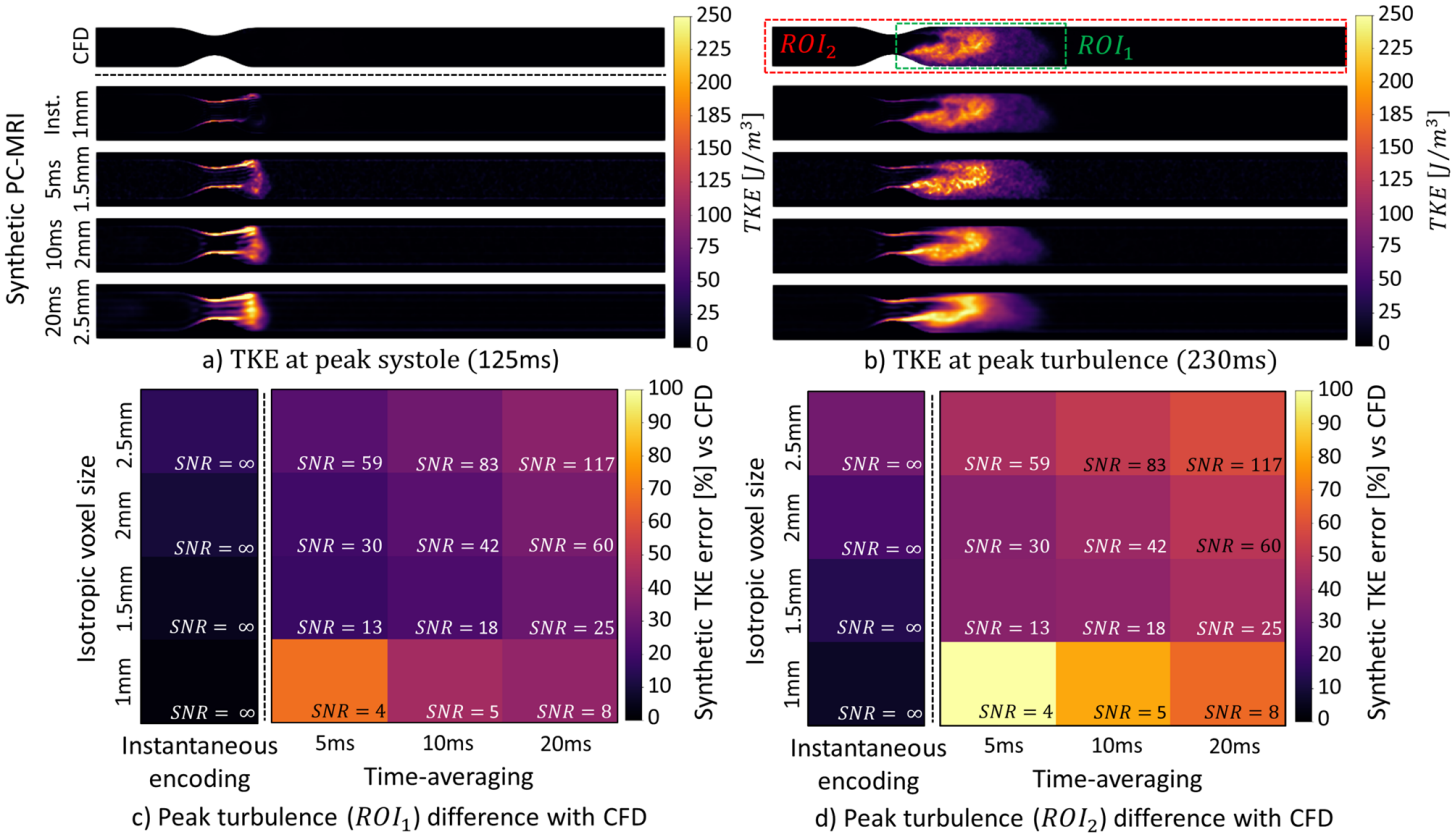
Impact of SNR and spatiotemporal resolution on turbulent kinetic energy for a 75% eccentric stenosis and pulsatile flow. Turbulent kinetic energy at peak systole (**a**) and at peak total TKE (**b**) is shown. Percentage errors of peak total TKE are compared in (**c**) and (**d**) for $RO{I}_{1}$ (envelope of the turbulent region) and $RO{I}_{2}$ (whole geometry). Instantaneous (Inst.) encoding refers to a hypothetical noise-free PC-MRI experiment with infinitely high velocity encoding bandwidth.

Figure 3 shows the effect of spatial resolution and time averaging on TKE quantification for pulsatile flow. For isotropic voxels sizes between 1 and $2.5\;\text{mm}$, total KE at peak systole is underestimated by up to 10% with hypothetical instantaneous encoding and by up to 22% when time averaging of $20\;\text{ms}$ is assumed. A delay between peak systole and maximum total TKE is seen in the simulated data. Figure 3a shows that temporal and spatial gradients artificially contribute to up to 100% of measured TKE. This effect is also visible to a lesser extent at peak TKE in Fig. 3b, where up to 40% of the measured total TKE is erroneous. For instantaneous encoding with isotropic voxel sizes between 1 and $2.5\;\text{mm}$, total TKE is overestimated by up to 15% and 31% for ${\mathrm{ROI}}_{1}$ (Fig. 3c) and ${\mathrm{ROI}}_{2}$ (Fig. 3d), respectively. With $2.5\;\text{mm}$ resolution and time averaging of $20\;\text{ms}$, total TKE is overestimated by up to 38% for ${\mathrm{ROI}}_{1}$ and 58% for ${\mathrm{ROI}}_{2}$.

**Figure 4:**
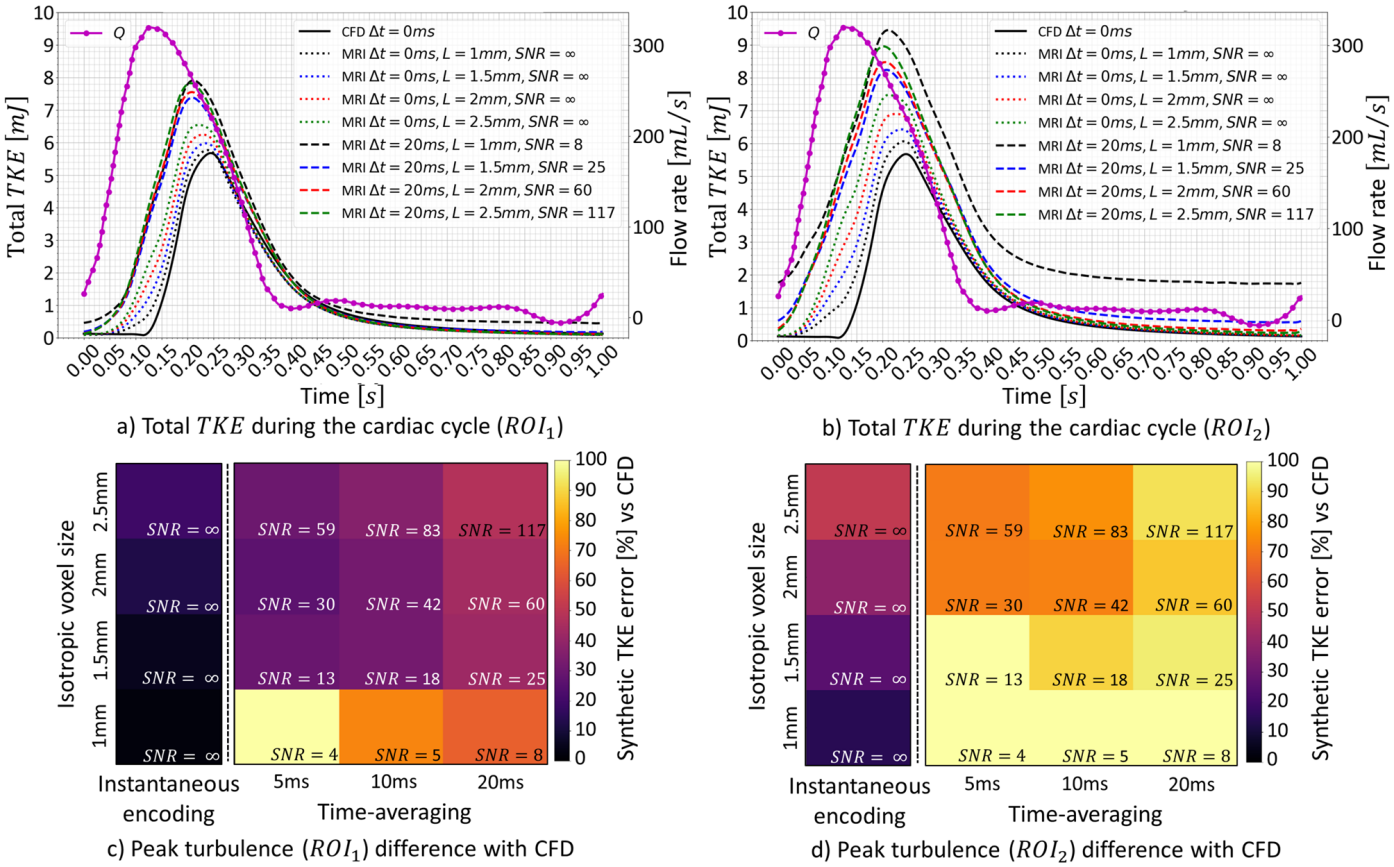
Impact of SNR and spatiotemporal resolution on time-resolved turbulent kinetic energy for a 75% eccentric stenosis and pulsatile flow. Time-resolved total TKE and flow rate $\left(Q\right)$ during a simulated cardiac cycle for varying voxel sizes $\left(L\right)$, time averaging $\left(\Delta t\right)$ and, correspondingly, signal-to-noise ratios $\left(SNR\right)$ for $RO{I}_{1}$ (**a**) and $RO{I}_{2}$ (**b**) (see Fig. 3). Errors in total TKE integrated during the cardiac cycle $RO{I}_{1}$ (**c**) and $RO{I}_{2}$ (**d**). Instantaneous ($\Delta t=0\;\text{ms}$) encoding refers to a hypothetical noise-free PC-MRI experiment with infinitely high velocity encoding bandwidth.

Figure 4a,b compares total TKE during a simulated cardiac cycle for different settings of spatial resolution, time averaging and SNR. Figure 4c,d visualize the effect of spatial resolution and time averaging on the quantification of total TKE integrated over the cardiac cycle. Isotropic spatial resolutions between $1.5$ and $2\;\text{mm}$ and time averaging of $5\;\text{ms}$ lead to overestimation of total TKE by up to 26% for ${\mathrm{ROI}}_{1}$ (Fig. 4c) and 70% for ${\mathrm{ROI}}_{2}$ (Fig. 4d), respectively.

### Patient-specific velocity and turbulence fields

**Figure 5:**
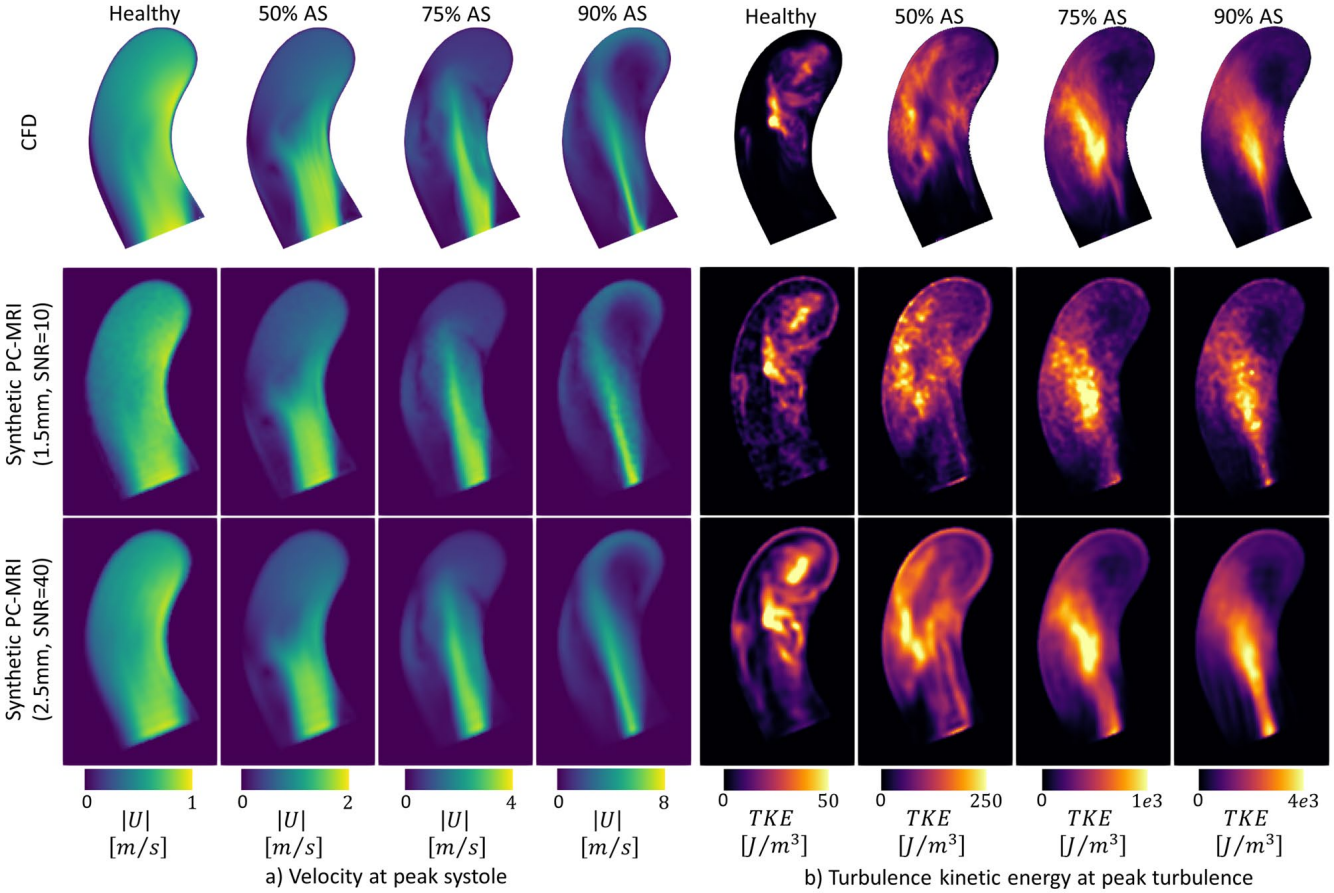
Patient-specific velocity and TKE maps for varying degrees of stenosis (foot-head slices of the aorta aligned with the inflow jet). (**a**) Magnitude of the velocity at peak systole and (**b**) turbulent kinetic energy at peak total TKE for two resolutions and the corresponding reference CFD. For (**a**) and (**b**), from left to right, healthy inlet flow and simulated stenosis degrees of 50%, 75% and 90% are shown. Note the difference in color bar scaling for both velocity and TKE depending on the stenosis degree. A video showing all time steps is available in the online supplemental material.

Figure 5 compares velocity magnitude and TKE maps at peak systole and peak TKE for CFD and synthetic PC-MRI. Artificially high TKE values are visible at the walls and in flow regions with high velocity gradients.

**Figure 6:**
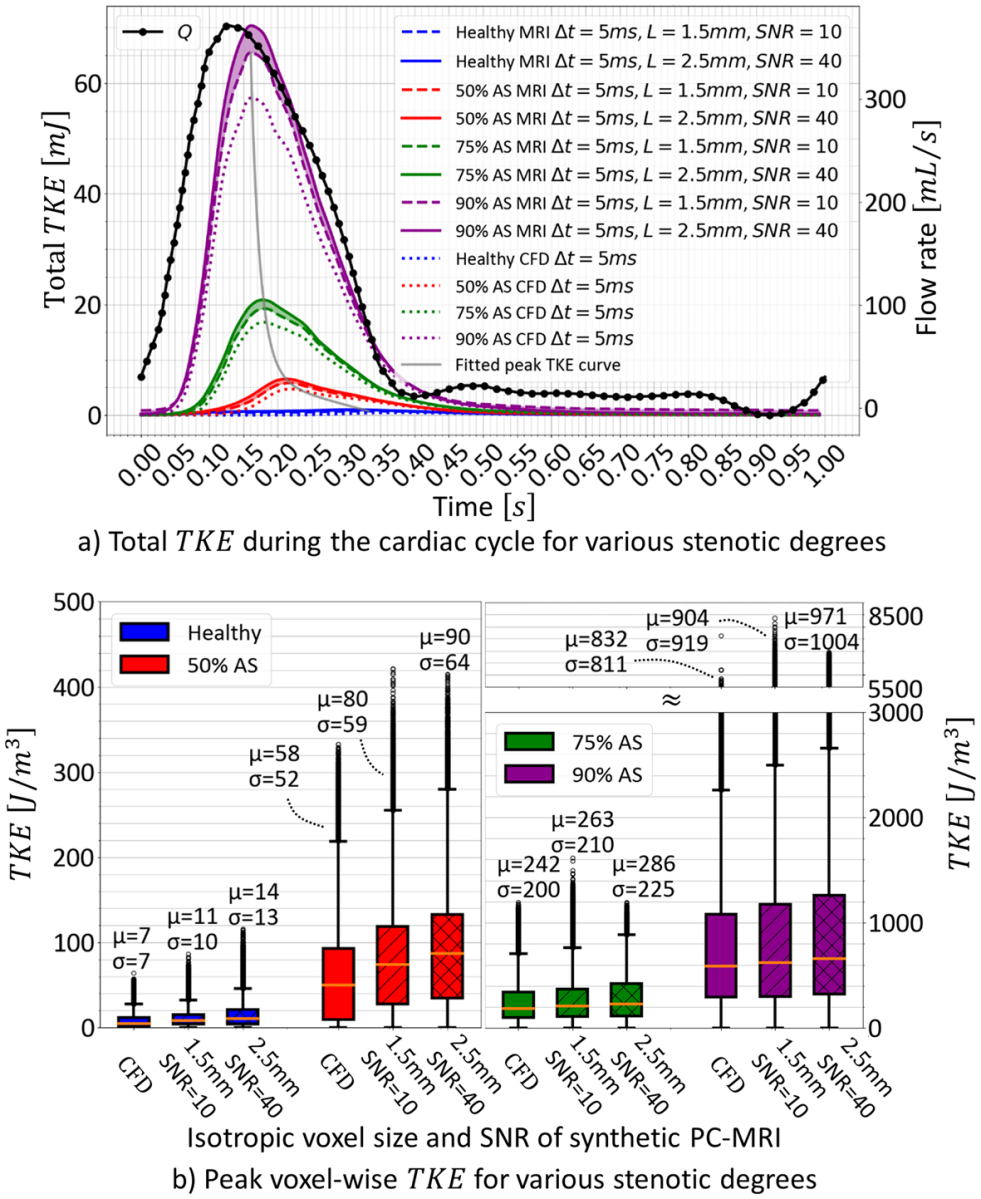
Variations in turbulent kinetic energy depending on the stenosis degree during the cardiac cycle and at peak total TKE. (**a**) Comparison of measured total TKE for two resolutions of PC-MRI and the reference CFD during the cardiac cycle for a healthy inlet flow and simulated aortic stenosis degrees of 50%, 75% and 90%. Note the temporal lag between peak flow rate and peak TKE represented by the fitted peak TKE curve. (**b**) Mean $\mu$ and standard deviation $\sigma$ of peak voxel-wise TKE for the two resolutions of PC-MRI for healthy flow and 50%, 75% and 90% stenoses.

Figure 6a shows the evolution of TKE for various degrees of stenosis during the cardiac cycle. Peak TKE occurs after peak systole with a delay $\delta$ that depends on the stenosis degree. For stenotic degrees of 50%, 75% and 90% the delay $\delta$ is 32, 53 and 90 ms. For mild to severe aortic stenosis, peak total TKE varies from $7$ to $70\;\text{mJ}$. Figure 6b summarizes peak TKE statistics.

Mean and standard deviation of TKE in the ascending aorta for stenotic degrees of 50, 75 and 90% are overestimated by 37.9, 8.6 and 8.6% and 13.5, 5.0 and 13.3%, respectively, for a voxel size of $1.5\;\text{mm}$. Similarly, the overestimation is 55.2, 18.2 and 16.7% and 23.1, 12.5 and 23.8% for a voxel size of $2.5\;\text{mm}$ (Fig. 6b). Higher spatial resolution results in outliers with larger TKE.

The patient-specific datasets presented in this work required on average 60 wall-clock hours using 48 cores to obtain the CFD solution, each time frame was around 260 MB.

## Discussion

In this study, a framework for the synthesis of time-resolved multipoint 4D flow MRI data of turbulent flow in patient-specific, moving aortic geometries has been presented. The impact of spatial resolution, time averaging and SNR was investigated for both steady and pulsatile flows in idealized geometries for a fixed hypothetical scan time budget.

The qualitative comparison of velocity and TKE maps in Fig. 2a,b–d confirmed that turbulence measurements are more sensitive to SNR and spatial resolution when compared to velocity measurements and that the ROI used to compute total TKE should be selected carefully. In Fig. 2c,d, limited spatial resolution and low SNR both contribute to overestimation of TKE due to contributions by noise and spatial velocity gradients in accordance with previous studies^35,36^. Variations in total TKE for different time averaging in Fig. 2c,d are due to SNR differences, as time averaging has no influence for steady flows.

Contributions by temporal velocity gradients further increase the artificial overestimation of TKE in pulsatile flows as demonstrated in Fig. 3a. Turbulence only appears after peak systole in the ground truth, suggesting TKE production is kick-started by post-systolic flow deceleration (Fig. 4a).

Spatial and temporal gradients compromise measured velocity and TKE values in voxels due to partial volume effects, which are visible at the walls of the aorta, where artificially high TKE values are present. In particular, we have demonstrated that, at peak systole, the measured RST is purely artificial and does not represent turbulence (Fig. 3a). Additionally, we have observed that peak TKE occurs with a delay compared to peak systole, suggesting that TKE quantification at peak systole as proposed in the literature may need to be reconsidered^33^.

For spatial resolutions of 1.5 and 2.5 mm and time averaging of 5 ms, peak total TKE downstream of a 75% stenosis is overestimated by 15 and 24%, respectively (Fig. 6a). Similar overestimations (18 and 25%) were observed using the idealized geometry with pulsatile flow, suggesting that observations in Figs. 2, 3 and 4 can be extrapolated to more realistic geometries and flows. Higher spatial resolution creates outliers with larger TKE values due to lower SNR levels. For a typical 4D flow MRI isotropic spatial resolution of $2.5\;\text{mm}$ and stenotic degree > 75%, voxel-wise TKE is consistently overestimated in the ascending aorta, suggesting that 4D flow MRI overestimation of TKE might be predictable for high turbulence regimes.

In the present study, net flow was identical for the different degrees of simulated stenoses. Since pressure along the aorta depends on the stenotic degree, variations in vessel cross-sectional area are expected, which were not modeled in our work. Future work is warranted to integrate wall displacements resulting from the interaction of flow and wall compliance to arrive at realistic pulse wave velocities. Full fluid–structure interaction approaches, or reduced order models applied to the elastic and viscoelastic response of the tissue could be translated from our previous works^37–40^. Also, anatomical variability could be augmented using anatomical models based on low rank reconstructions^41^.

Another limitation of the present study relates to the use of a simplified approach for generating PC-MRI signals, assuming ideal encoding and readout and, therefore, neglecting the impact of encoding schemes on measured values of velocity and turbulence. Flow-induced displacement artifacts were not included, and cycle-to-cycle variations were condensed into cycle-averaged quantities. These assumptions were made to reduce the computational cost as compared to more accurate modelling of the imaging processes^31^. Recent work from Dillinger et al.^42^ demonstrates that the implementation of realistic encoding gradients in an Eulerian–Lagrangian Bloch solver results in systematic underestimation of high frequency components of turbulence. Therefore, quantification of in-vivo turbulence is subject to overestimation due to partial volume effects on one hand and underestimation due to band-limited velocity encoding gradients on the other hand.

The study also lacks comparison between in-vivo and synthetic 4D flow MRI datasets. However, due to the complexity of flow patterns in the aorta and assumptions used in patient-specific CFD, only a qualitative comparison of flow patterns would be achievable^26,43,44^. Although this is a general limitation for patient-specific simulations, it does not directly affect our proposed workflow as our aim is to generate realistic flows in the aorta rather than to attempt to duplicate patient-specific hemodynamics in every detail.

To conclude, the synthesis framework presented here enables the generation of paired sets of patient-specific realistic ground truth and 4D flow MRI data to cater to training of deep learning algorithms for image reconstruction^15^ and inference^21^ in the future. Although this work focused on velocity and TKE in post-stenotic aortic flows, the ground truth CFD contains information about pressure drop, wall shear stresses and pulse wave velocity, opening the door to the possibility of analyzing other important hemodynamic biomarkers in future works. Additionally, the patient-specific nature of this work suggests that, if adequate datasets are available, the pipeline could be used to generate synthetic 4D flow datasets for other valvular or aortic pathologies, such as aortic regurgitation (AR), bicuspid aortic valve (BAV) and dilated ascending aorta.

## Methods

### Idealized computational domain

An eccentric stenotic tube with fixed walls and stenosis severity of 75%^45,46^ was modeled as idealized geometry. This geometry has been widely studied in the literature^34,47,48^ and has an analytical description with the stenosis modelled as a cosine function offset in one direction by an eccentricity of 5% of the diameter. The stenosis throat was positioned at a distance of 3 diameters from the inlet, and the cylinder extended for 20 diameters downstream. A structured hexahedral butterfly mesh with 2.7 M cells was generated using OpenFoam’s blockMesh utility^49^.

### Patient-specific computational domain

Realistic aortic geometries were obtained from in-vivo MRI data. The subjects were studied upon written informed consent under the approval of the ethics committee of the Canton of Zurich, Switzerland, and according to institutional guidelines. Imaging experiments were performed on a 1.5 T MR system (Philips Healthcare, Best, The Netherlands) using a 32-channel receive array. High-resolution cine balanced steady-state free precession slices ($1\times 1\times 5\;\text{mm}^{3}$) were acquired orthogonally to the aorta centerline with a temporal resolution of 40 frames/cardiac cycle in a breath hold. A total of 9 slices were distributed uniformly along the aortic center line, covering the aortic arch and the descending aorta with the first slice positioned at the aortic root. Lumen boundaries were extracted and the corresponding 3D surface extrapolated from segmented 2D contours (Supplemental Fig. S1). The brachiocephalic, left common carotid and left subclavian arteries were removed, as their impact on flow features in the ascending aorta is not significant^50^. A structured hexahedral butterfly mesh was then generated using OpenFoam’s blockMesh utility^49^ on the anatomy at end diastole, which was considered as the initial phase of the cardiac cycle (Fig. 1a). A mesh size of 2.2 M cells with mean and maximum cell heights in the region of interest of $0.37\;\text{mm}$ and $0.6\;\text{mm}$ was found to be sufficient to accurately simulate both velocity and turbulence fields in aortae with pathological inflows^51–53^.

### Boundary conditions

Fully developed Hagen-Poiseuille profiles were used as inlet boundary conditions for the idealized geometries. The mean inlet Reynolds number $(Re)$ was set to 1000 for both steady and pulsatile scenarios. For the pulsatile case, the velocity waveform was extracted from in-vivo data acquired at the aortic root in a healthy subject with a peak inlet $Re$ of 4000, typical for physiological flows^54^.

Aortic wall motion for the patient-specific simulation was extracted for all cardiac phases and was used as boundary condition for the CFD simulation. Time-resolved inlet velocity profiles for the patient-specific simulations were extracted from time-resolved 2D PC MRI spoiled gradient echo imaging ($1.5\times 1.5\times 8 \;\text{mm}^{3}$, 40 frames/cardiac cycle). Pathological stenotic inlets were generated by projecting the healthy inlet velocities onto reduced cross sections (50, 75 and 90%) of the geometrical model inlet while keeping the flow rate constant (Supplemental Fig. S2).

### Computational fluid dynamics

Blood flow in the aorta was computed using the three-dimensional, unsteady and incompressible Navier–Stokes (NS) equations in moving domains. Blood was assumed Newtonian and incompressible with density $\rho =1060 \;\text{kg}/\text{m}^{3}$ and kinematic viscosity $\mu =3.5{e}^{-3} \; \text{Pa} \,\text{s}$^55^. In our work, the NS equations were solved using a large eddy simulation (LES) model in the arbitrary Lagrangian–Eulerian (ALE) framework as implemented in OpenFOAM^®^ v1806^49^. The subgrid scheme selected was the wall-adapting local-eddy viscosity (WALE) subgrid-scale (SGS) model^51,52^ and Spalding’s wall function was used^56^. Second-order central differences and backward Euler schemes were used for spatial and temporal discretization. Adaptive time stepping was used to reduce simulation times; at peak turbulent production the time step ranged between 25 and 100 μs depending on the stenotic degree^7,34^ (Fig. 1b). Simulations were run on a high-performance cluster of the Swiss National Supercomputing Center (CSCS). On average 6 wall clock hours per cardiac cycle were required using 48 cores (300 CPU hours) for a simulation of moderate aortic stenosis.

### Computation of the Reynolds stress tensor

The covariance matrix $Cov({\varvec{u}})$ of $N$ measurements of a time varying velocity vector ${\varvec{u}}=({{\varvec{u}}}_{1},{{\varvec{u}}}_{2},\ldots ,{{\varvec{u}}}_{{\varvec{N}}})\in {\mathbb{R}}^{3\times N}$ is defined as:

1 $$Cov\left({\varvec{u}}\right)=\frac{1}{N}\sum_{n=1}^{N}\left({{\varvec{u}}}_{{\varvec{n}}}-\overline{{\varvec{u}} }\right){\left({{\varvec{u}}}_{{\varvec{n}}}-\overline{{\varvec{u}} }\right)}^{\mathrm{T}}=\overline{{{\varvec{u}} }^{\boldsymbol{^{\prime}}}{{{\varvec{u}}}^{\boldsymbol{^{\prime}}}}^{{\varvec{T}}}}={\rho }^{-1}{\varvec{R}}$$

where $\bar{\blacksquare}$ is the averaging operator, ${{\varvec{u}}}^{\boldsymbol{^{\prime}}}={\varvec{u}}-\overline{{\varvec{u}} }$ are the velocity fluctuations over the mean velocity $\overline{{\varvec{u}} }$ , ${{\varvec{u}}}^{\boldsymbol{{\prime}}}{{{\varvec{u}}}^{\boldsymbol{{\prime}}}}^{{\varvec{T}}}={{\varvec{u}}}^{\boldsymbol{{\prime}}} \otimes {\boldsymbol{ }{\varvec{u}}}^{\boldsymbol{{\prime}}}$ defines an outer product, and ${\varvec{R}}\in {\mathbb{R}}^{3\times 3}$ is the Reynolds stress tensor. In the case of pulsatile flow, the $N$ measurements $\left({{\varvec{u}}}_{1},{{\varvec{u}}}_{2},\dots ,{{\varvec{u}}}_{{\varvec{N}}}\right)$ are acquired at the same time ${t}_{0}$ of the cycle, over $N$ cycles. Due to band-limited encoding and finite readout times in MRI, flow measurements are not instantaneous snapshots, but include flow information over finite durations. To model this condition, measurements of $\overline{{\varvec{u}} }$ and ${\varvec{R}}$ are performed in a temporal window $\Delta t$ around ${t}_{0}$, where $\Delta t$ corresponds to the modeled temporal averaging duration of the acquisition.

### Synthetic 4D flow MRI

The MR signal ${S}^{*}$, assuming Gaussian intra-voxel velocity distribution (IVSD) of variance ${\sigma }_{{k}_{v}}$ reads (Fig. 1d):

2 $${S}^{*}\left({{\varvec{k}}}_{{\varvec{v}},{\varvec{i}}}\right)={S}_{0}{\mathrm{e}}^{-\frac{{\sigma }_{{k}_{v},i}^{2}{\left|{{\varvec{k}}}_{{\varvec{v}},{\varvec{i}}}\right|}^{2}}{2}}{\mathrm{e}}^{-j{{\varvec{k}}}_{{\varvec{v}},{\varvec{i}}}{\overline{{\varvec{u}}} }_{{\varvec{\Delta}}}}+\eta$$

where ${{\varvec{k}}}_{{\varvec{v}},{\varvec{i}}}={{k}_{v,i}{\overrightarrow{{\varvec{e}}}}_{i}=\left[{k}_{vx},{k}_{vy},{k}_{vz}\right]}_{i}\in {\mathbb{R}}^{1\times 3}$ represents flow sensitivity along the ${i}{\mathrm{th}}$ direction with encoding velocity frequency ${k}_{v,i}=\pi /[\mathrm{VEN}{\mathrm{C}]}_{i}.$ $\eta \propto \mathrm{SNR}$ is complex Gaussian noise with zero mean and standard deviation ${\sigma }_{\eta }={\left|{\overline{S} }_{ROI}\right|\cdot \left(\mathrm{SNR}\right)}^{-1}$ with ${\overline{S} }_{ROI}$ being the mean noise-free signal in the region of interest, defined as the full fluid domain for all simulations. ${S}_{0}$ is the normalized reference signal without velocity encoding that in this work is modelled as:

3 $${S}_{0}=\frac{1}{2}\left[{\left(\frac{\left|{\overline{{\varvec{u}}} }_{{\varvec{\Delta}}}\right|}{{\left|{\overline{{\varvec{u}}} }_{{\varvec{\Delta}}}\right|}_{max}}\right)}^\frac{1}{3}+1\right]$$

where ${\overline{{\varvec{u}}} }_{{\varvec{\Delta}}}$ is the velocity field at the selected MR signal resolution. The term ${\sigma }_{{k}_{v},i}^{2}{\left|{{\varvec{k}}}_{{\varvec{v}},{\varvec{i}}}\right|}^{2}$ can be expressed as ${{\rho }^{-1}{\varvec{k}}}_{{\varvec{v}},{\varvec{i}}}{{\varvec{R}}}_{{\varvec{\Delta}}}^{{\varvec{t}}}{{{\varvec{k}}}_{{\varvec{v}},{\varvec{i}}}}^{\mathrm{T}}$ where ${{\varvec{R}}}_{{\varvec{\Delta}}}^{{\varvec{t}}}$ is the Reynolds stress tensor at the selected MR resolution ${\Delta }_{\mathrm{L}}$. Both ${{\varvec{R}}}_{{\varvec{\Delta}}}^{{\varvec{t}}}$ and ${\overline{{\varvec{u}}} }_{{\varvec{\Delta}}}$ are obtained by first projecting computed values from the CFD simulations onto a regular grid $({n}_{x}\times {n}_{y}\times {n}_{z})$ with isotropic voxel size $L=0.65\;\text{mm}$. The fields are subsequently downsampled to the prescribed MR resolution, ${\Delta }_{\mathrm{L}}$, by apodization with a truncated 3D Gaussian modular transfer function (MTF) $\omega$ with standard deviation ${\sigma }_{G}=\sqrt{8\mathrm{ln}2}L/{\Delta }_{\mathrm{L}}$. The truncation window is a box with width $w\propto L/{\Delta }_{\mathrm{L}}$, such that the Gaussian MTF is truncated at an amplitude of 0.5 along each principal Cartesian direction. The downsampled RST ${{\varvec{R}}}_{{\varvec{\Delta}}}^{{\varvec{t}}}$ and velocity ${\overline{{\varvec{u}}} }_{{\varvec{\Delta}}}$ are then defined as (Fig. 1c.1 and c.2):

4 $${\overline{{\varvec{u}}} }_{{\varvec{\Delta}}}={\mathcal{F}}^{-1}\left(\mathcal{F}\left(\overline{{\varvec{u}} }\right)\circ \omega \right)$$

5 $${{\varvec{R}}}_{{\varvec{\Delta}}}^{{\varvec{t}}}={\mathcal{F}}^{-1}\left(\mathcal{F}\left({{\varvec{R}}}^{{\varvec{t}}}\right)\circ \omega \right)+\rho {\mathcal{F}}^{-1}\left(\mathcal{F}\left(\overline{{\varvec{u}} }{\overline{{\varvec{u}}} }^{{\varvec{T}}}\right)\circ \omega \right)-\rho {\overline{{\varvec{u}}} }_{{\varvec{\Delta}}}{\overline{{\varvec{u}}} }_{{\varvec{\Delta}}}^{{\varvec{T}}}$$

where $\mathcal{F}$ is the Fourier operator and $\circ$ is the apodization operator. Synthetic noise was defined for the idealized geometries (Figs. 2, 3 and 4) as a function of voxel volume $V$ and temporal averaging of signal $\Delta t$ (analogous to the repetition time $TR$ assuming that signal is continuously acquired during this time) as $\mathrm{SNR}=\alpha V\sqrt{\Delta t}$, where $\alpha =1.68$ is a scaling factor designed to obtain $SNR=30$ for $V=2\times 2\times 2 \;\text{mm}^{3}$ and $\Delta t=5\;\text{ms}$.

### Reynolds stress tensor reconstruction

The RST can be determined by encoding along six non-collinear directions and solving a system of linear equations. For six measurements along six different velocity encoding directions $\left\{i \right| i\in {\mathbb{Z}},1\le i\le 6\}$, ${\sigma }_{{k}_{v},i}$ is obtained from the ratio between ${S}^{*}\left({{\varvec{k}}}_{{\varvec{v}},{\varvec{i}}}\right)$ and ${S}^{*}\left(0\right)$ as:

6 $${\sigma }_{{k}_{v},i}^{2}=\frac{2}{{\left|{{\varvec{k}}}_{{\varvec{v}},{\varvec{i}}}\right|}^{2}}\mathrm{ln}\frac{\left|{S}^{*}\left(0\right)\right|}{\left|{S}^{*}\left({{\varvec{k}}}_{{\varvec{v}},{\varvec{i}}}\right)\right|}\approx \frac{{{{\varvec{k}}}_{{\varvec{v}},{\varvec{i}}}}^{{\varvec{T}}}{{\varvec{R}}}^{\boldsymbol{*}}{{\varvec{k}}}_{{\varvec{v}},{\varvec{i}}}}{{\rho \left|{{\varvec{k}}}_{{\varvec{v}},{\varvec{i}}}\right|}^{2}}$$

where ${{\varvec{R}}}^{\boldsymbol{*}}$ is the estimated RST. By rewriting Eq. (6), the following system of linear equations is obtained:

7 $${\sigma }_{{k}_{v}.i}^{2}=\frac{1}{{\rho \left|{{\varvec{k}}}_{{\varvec{v}},{\varvec{i}}}\right|}^{2}}({k}_{vx,i}^{2},{k}_{vy,i}^{2},{k}_{vz,i}^{2},2{k}_{vx,i}{k}_{vy,i},2{k}_{vx,i}{k}_{vz,i},2{k}_{vy,i}{k}_{vz,i})\cdot {\left({R}_{xx},{R}_{yy},{R}_{zz},{R}_{xy},{R}_{xz},{R}_{yz}\right)}^{T}={{\varvec{H}}}_{{\varvec{i}}}\left({{\varvec{k}}}_{{\varvec{v}},{\varvec{i}}},\rho \right){{\varvec{r}}}^{\boldsymbol{*}}$$

where ${{\varvec{\sigma}}}_{{{\varvec{k}}}_{{\varvec{v}}}}$ $\in {\mathbb{R}}^{6\times 1}$ is the IVSD vector, ${{\varvec{H}}}_{{\varvec{i}}}\left({{\varvec{k}}}_{{\varvec{v}},{\varvec{i}}},\rho \right)$ is the ${i}{\mathrm{th}}$ row of ${\varvec{H}}\left({{\varvec{k}}}_{{\varvec{v}}},\rho \right)$ $\in {\mathbb{R}}^{6\times 6}$**,** a transformation matrix that depends on ${{\varvec{k}}}_{{\varvec{v}}}\in {\mathbb{R}}^{6\times 3}$ and $\rho$, and ${{\varvec{r}}}^{\boldsymbol{*}}$ $\in {\mathbb{R}}^{6\times 1}$ is the vector representation of the symmetric RST tensor ${{\varvec{R}}}^{\boldsymbol{*}}$. The encoding matrix ${{\varvec{k}}}_{{\varvec{v}}}$ was designed for orthogonal encoding^35,57^ in this study, but can be modified for any other encoding scheme. The elements of the RST can be calculated voxel-wise using the pseudoinverse (Fig. 1f):

8 $${{\varvec{r}}}^{\boldsymbol{*}}={\left({{\varvec{H}}}^{\mathrm{T}}{\varvec{H}}\right)}^{-1}{{\varvec{H}}}^{\mathrm{T}}{{\varvec{\sigma}}}_{{{\varvec{k}}}_{{\varvec{v}}}}^{2}$$

The symmetric RST vector ${{\varvec{r}}}^{\boldsymbol{*}}$ can be recast into its tensor representation ${{\varvec{R}}}^{\boldsymbol{*}}\in {\mathbb{R}}^{3\times 3}$. The elements along the diagonal represent velocity fluctuation variances while the off-diagonal elements represent covariances. Turbulent kinetic energy in [J/m^3^] is then defined as:

9 $$\mathrm{TKE}=1/2\;\mathrm{Tr}\left({{\varvec{R}}}^{\boldsymbol{*}}\right)$$

where $\mathrm{Tr}\left({{\varvec{R}}}^{\boldsymbol{*}}\right)$ is the trace of the RST. Total TKE in [mJ] refers to the volumetric integration of TKE in a region of interest.

### Velocity reconstruction

Redundant encoding schemes provide additional information for estimation of mean velocities. The velocities encoded in the six directions are defined by ${\mathop{\varvec{\nu}}\limits^{\smile}}=\mathrm{arg}\left({S}^{*}\left({{\varvec{k}}}_{{\varvec{v}},{\varvec{i}}}\right)\right)\in {\mathbb{R}}^{6\times 1}$ and can be written as:

10 $${\mathop{\nu}\limits^{\smile}}_{i}={{\varvec{k}}}_{{\varvec{v}},{\varvec{i}}}{\left|{{\varvec{k}}}_{{\varvec{v}},{\varvec{i}}}\right|}^{-1}{{\varvec{u}}}^{\boldsymbol{*}}={{\varvec{K}}}_{{\varvec{i}}}{\left({{\varvec{k}}}_{{\varvec{v}},{\varvec{i}}}\right){\varvec{u}}}^{\boldsymbol{*}}$$

where ${{\varvec{K}}}_{{\varvec{i}}}\left({{\varvec{k}}}_{{\varvec{v}},{\varvec{i}}}\right)$ is the ${i}{\mathrm{th}}$ row of ${\varvec{K}}\left({{\varvec{k}}}_{{\varvec{v}}}\right)\in {\mathbb{R}}^{6\times 3}$, the normalized encoding tensor and ${{\varvec{u}}}^{\boldsymbol{*}}\in {\mathbb{R}}^{3\times 1}$ is the Cartesian velocity vector. A solution to this overdetermined system of linear equations is provided by the pseudo-inverse (Fig. 1f):

11 $${{\varvec{u}}}^{\boldsymbol{*}}={\left({{\varvec{K}}}^{\mathrm{T}}{\varvec{K}}\right)}^{-1}{{\varvec{K}}}^{\mathrm{T}}{\mathop{\varvec{\nu}}\limits^{\smile}}$$

From the Cartesian velocity vector ${{\varvec{u}}}^{\boldsymbol{*}}$, kinetic energy (KE) in [J/m^3^] is defined as:

12 $$\mathrm{KE}=\frac{\rho }{2}{\left|{{\varvec{u}}}^{\boldsymbol{*}}\right|}^{2}$$

Total KE in [mJ] refers to the volumetric integration of KE in a region of interest.

### Bayesian reconstruction

Turbulence estimation shows high sensitivity within a limited range of IVSD values dictated by the choice of velocity encoding (VENC) and, respectively, encoding strength ${k}_{v}=\pi /\mathrm{VENC}$. This suggests that single VENC acquisitions are limited in their ability to probe the rich variety of expected IVSD in pathological aortic flows^12^. To mitigate this effect, a multipoint approach was used to probe velocity and turbulence fields using orthogonal encoding with three different encoding strengths^35^ (Supplemental Table S1). For each encoding direction, the acquisitions at different encoding strengths were combined using Bayesian multipoint unfolding^12^ to generate a set of directional velocities ${\mathop{\nu}\limits^{\smile}}_{i}$ and IVSD ${\sigma }_{{k}_{v} \cdot i}$ that were then converted to velocities and RST using Eqs. (8 and 11).

## Supplementary Information

Supplementary Information.

Supplementary Video 1.

Supplementary Video 2.

## Data Availability

Our Python code for 4D flow MRI synthesis and Bayesian reconstruction is publicly available (https://gitlab.ethz.ch/ibt-cmr-public/4dflowmrisynthesis), accompanied with demo data corresponding to the idealized geometry with pulsatile flow presented in Fig. 3b).
