# Supplementary material for: Synthesis of patient-specific multipoint 4D flow MRI data of turbulent aortic flow downstream of stenotic valves
Source: Sci Rep. 2022 Sep 26;12:16004. doi: 10.1038/s41598-022-20121-x (PMC9513106; doi:10.1038/s41598-022-20121-x)
Supplement: Supplementary file 1 — Supplementary Information. [file 41598_2022_20121_MOESM1_ESM.pdf]

Synthesis of Patient-Specific Multipoint 4D Flow MRI Data of Turbulent Aortic Flow Downstream of Stenotic Valves: Supplementary Material

Pietro Dirix*, Stefano Buoso, Eva S. Peper & Sebastian Kozerke

Institute for Biomedical Engineering, University and ETH Zurich, Zurich, Switzerland

Supplementary Figures

Time-resolved segmentation of the aorta

In Fig. S1, the patient-specific computational domain and boundary conditions discussed in the Methods section are visualized. Four slices along the aorta are displayed with their corresponding CFD velocity and 2D cine MRI data during systole as well as their area variation during the cardiac cycle. The patient-specific velocity profile extracted from a 2D PC-MRI slice is imposed at the model inlet and presents the characteristic velocity profile of a healthy tricuspid valve. The area variation measured from the cine data is imposed at the walls.

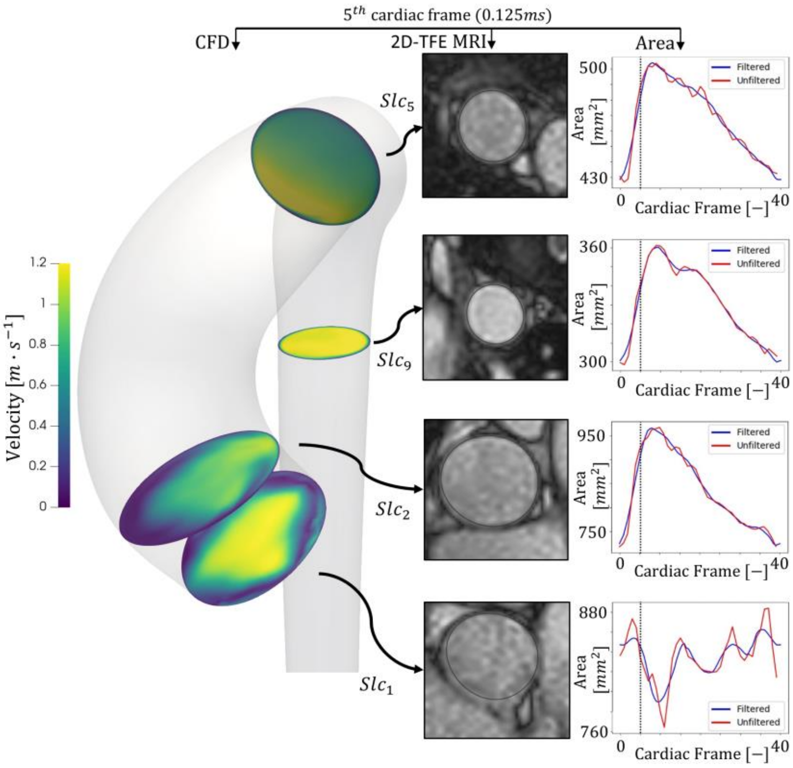

Fig. S1: Visualization of reconstructed CFD geometry and corresponding 2D cine MRI slices for the 5th acquired cardiac frame (peak systole). Note that the segmented area at the inlet of the geometry (Slc_1) does not follow the expected behavior, which is related to through-plane motion of the aortic root. Slices Slc_3, Slc_4, Slc_6, Slc_7 and Slc_8 were omitted for clarity, but all 9 slices were used to generate the transient CFD geometry. After Slc_9 the aorta was extended to ensure convergence of CFD simulation. A video showing all time steps is available in the online supplemental material.

Patient-specific inflow

In Fig. S2, the patient-specific velocity boundary condition discussed in the Methods section is represented. A 2D PC-MRI scan of the aortic inlet is segmented and the used to extract the time-resolved patient-specific velocity profile. Five time-steps are presented with their corresponding normalized axial velocities and the projection of the extracted velocity profile onto the model. Increased stenotic degree was modeled by projecting the healthy inlet velocity profile onto reduced cross-sections of the geometrical model inlet while keeping the flow rate constant.

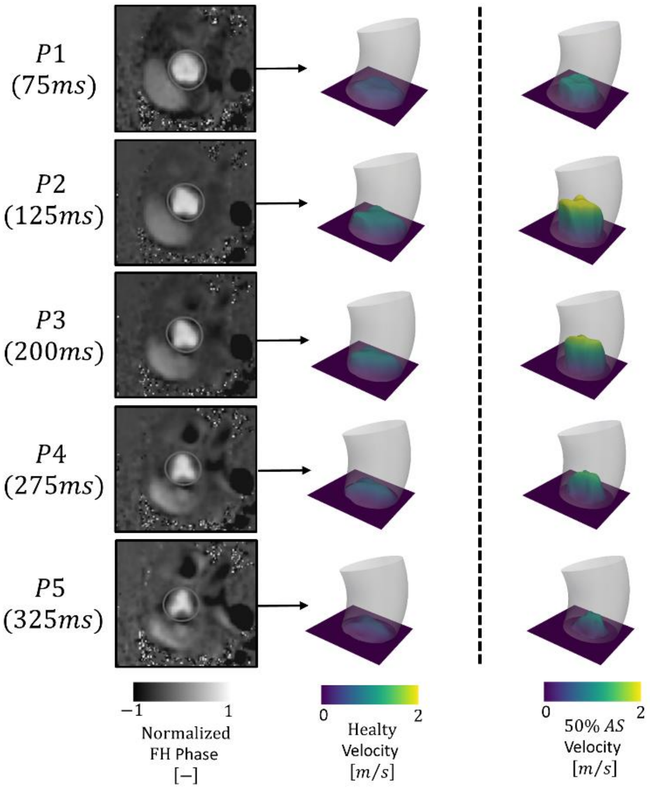

Fig. S2: Extraction of velocity at the aortic inlet from 2D PC-MRI images at different times in the cardiac cycle P1 (75ms), P2 (125ms), P3 (200ms), P4 (275ms) and P5 (325ms). Although only axial PC-MRI velocity data is presented here, coronal and sagittal velocity components were also used to reconstruct the 3D inlet velocity profile.

Supplementary Videos

Patient-specific velocity and TKE maps for varying degrees of stenosis

Video. S1: Patient-specific velocity and TKE maps for varying degrees of stenosis (foot-head slices of the aorta aligned with the inflow jet). (a) Magnitude of the velocity during the cardiac cycle and (b) turbulent kinetic energy during the cardiac cycle for two resolutions and the corresponding reference CFD. For (a) and (b), from left to right, healthy inlet flow and simulated stenosis degrees of 50%, 75% and 90% are shown. Note the difference in color bar scaling for both velocity and TKE depending on the stenosis degree.

Time-resolved segmentation of the aorta

Video. S2: Visualization of reconstructed CFD geometry and corresponding 2D cine MRI slices during the cardiac cycle. Note that the segmented area at the inlet of the geometry (Slc_1) does not follow the expected behavior, which is related to through-plane motion of the aortic root. Slices Slc_3, Slc_4, Slc_6, Slc_7 and Slc_8 were omitted for clarity.

Supplementary Methods

Details of the idealized stenotic geometry

The cross-stream coordinates y and z of the stenotic area in the idealized geometry used in this work are computed along the axial coordinate x by using the cross-section function $C(x)$ and the eccentricity function $E(x)$:

$$
\begin{gathered}
C(x) = \frac{D}{2}\left[1 - s_0\left(1 + \cos\left(\frac{2\pi x}{L}\right)\right)\right] \\
E(x) = \frac{s_0}{10}\left(1 + \cos\left(\frac{2\pi x}{L}\right)\right) \cdot D \\
y = S(x)\cos\theta \\
z = E(x) + S(x)\sin\theta
\end{gathered} \tag{1}
$$

where $D = 30mm$ is the diameter, $s_0 = 0.25$ represents the 75% area reduction at the stenotic throat and $L = 2D$ is the length of the stenosis $(-L/2 < x < L/2)$. The total geometry is obtained by extruding the pre- and post-stenosis boundaries.

Details on segmentation and mesh generation

A semi-automatic Python tool based on the random walker [1] and Chan-Vese [2] algorithms was developed to extract lumen boundaries from cine balanced steady-state free precession slices ($1 \times 1 \times 5\ mm^3$) acquired orthogonally to the aorta centerline. The brachiocephalic, left common carotid and left subclavian arteries were removed, as their impact on flow features in the ascending aorta is not significant [3]. Lumen boundaries were parametrized with 2D splines, radially resampled with $N_p = 500$ points and projected into 3D space. Then, sets of corresponding points between slices were identified and used to fit 4th order B-splines in order to generate a structured set of cloud points describing the surface of the aorta (Fig. S1).

Notes on the boundary conditions

Aortic meshes were extracted for all cardiac phases using the aforementioned approach. The motion of the aorta was computed by looking at the displacement between successive aortic meshes. Subsequently, aortic wall motion data was interpolated and upsampled to 80 frames/cardiac cycle using 3rd order B-splines. The boundary displacement field was enforced along the walls of the aorta (boundary condition for the CFD simulation) and was propagated to the internal mesh by solving a Laplace smoothing equation [4]. Time-resolved inlet velocity profiles were extracted from time-resolved 2D PC MRI spoiled gradient echo imaging acquired immediately downstream of the aortic valve. Velocity components were processed using anisotropic diffusion filtering [5], mapped to the inlet of the computational domain and upsampled to 80 frames/cycle using 3rd order B-splines.

During the simulation, both then inlet velocity field and mesh displacement field were linearly interpolated from the available frames, a temporal resolution of 80 frames/cycle allowed to obtain sufficiently smooth boundary conditions.

Derivation of the Reynolds stress tensor for the case of measurements with finite duration

In turbulent flow the velocity $\boldsymbol{u}$ can generally be decomposed in a mean term $\overline{\boldsymbol{u}}$ and a fluctuating term $\boldsymbol{u}'$ such that $\boldsymbol{u} = \overline{\boldsymbol{u}} + \boldsymbol{u}'$. The common definition of the Reynolds stress tensor in fluid dynamics is:

$$
\boldsymbol{R} = \rho\overline{\boldsymbol{u}'\boldsymbol{u}'^{\boldsymbol{T}}} = \frac{\rho}{N}\sum_{n=1}^{N}(\boldsymbol{u_n} - \overline{\boldsymbol{u}})\,(\boldsymbol{u_n} - \overline{\boldsymbol{u}})^{\mathrm{T}} \tag{2}
$$

where ρ is the fluid density and $\overline{\blacksquare}$ is an averaging operator. In the case of pulsatile flow, $\overline{\blacksquare}$ is typically a phase averaging operator (over N cardiac cycles) and $\boldsymbol{u}$ corresponds to an instantaneous measurement. However, due to band-limited encoding and finite readout times in MRI, flow measurements are not instantaneous snapshots, but rather include flow information over finite durations. To model this condition, it was assumed that for a given cycle n and a given selected phase in the cardiac cycle, t_0, M velocity measurements $\boldsymbol{u_n} = (\boldsymbol{u_{n,1}}, \boldsymbol{u_{n,2}}, \dots, \boldsymbol{u_{n,M}})$ are performed in a temporal window ΔT around t_0, where ΔT corresponds to the modeled temporal averaging duration and defines the temporal resolution of the acquisition. This was repeated over N cycles resulting in $\boldsymbol{u} = (\boldsymbol{u_{1,1}}, \boldsymbol{u_{2,1}}, \dots, \boldsymbol{u_{N,M}}) \in \boldsymbol{R}^{3\times(NM)}$. In the limit of $\Delta T \to 0$, $\boldsymbol{u}$ corresponds to the form used in Eq. (2). Assuming $\Delta T > 0$, the Reynolds stress tensor is:

$$\boldsymbol{R^t} = \rho \frac{1}{N \cdot M} \sum_{n=1}^{N} \sum_{m=1}^{M} (\boldsymbol{u_{n,m}} - \overline{\boldsymbol{u}})(\boldsymbol{u_{n,m}} - \overline{\boldsymbol{u}})^{\mathrm{T}} \tag{3}$$

where $N \cdot M$ measurements need to be stored. Let us consider $\boldsymbol{AB} = \boldsymbol{A} \cup \boldsymbol{B}$ the union of disjoint velocity partitions $\boldsymbol{A} = (\boldsymbol{v_a^1}, \boldsymbol{v_a^2}, \dots, \boldsymbol{v_a^h})$ and $\boldsymbol{B} = (\boldsymbol{v_b^1}, \boldsymbol{v_b^2}, \dots, \boldsymbol{v_b^q})$ with corresponding weights $w_A = h/(h+q)$ and $w_B = q/(h+q)$. A and B can be considered the velocity measurements of two cycles. If h and q are sufficiently large, then it is possible to compute $Cov(\boldsymbol{AB})$ solely from the statistics of the partitions [6]:

$$\begin{aligned} Cov(\boldsymbol{AB}) &= w_A \cdot Cov(\boldsymbol{A}) + w_B \cdot Cov(\boldsymbol{B}) + w_A(\overline{\boldsymbol{A}} - \overline{\boldsymbol{AB}})(\overline{\boldsymbol{A}} - \overline{\boldsymbol{B}})^{\mathrm{T}} \\ &\equiv w_A \cdot Cov(\boldsymbol{A}) + w_B \cdot Cov(\boldsymbol{B}) + w_A(\overline{\boldsymbol{A}} - \overline{\boldsymbol{AB}})(\overline{\boldsymbol{A}} - \overline{\boldsymbol{AB}})^{\mathrm{T}} \\ &+ w_B(\overline{\boldsymbol{B}} - \overline{\boldsymbol{AB}})(\overline{\boldsymbol{B}} - \overline{\boldsymbol{AB}})^{\mathrm{T}} \end{aligned} \tag{4}$$

where $\overline{\boldsymbol{AB}} = w_A\overline{\boldsymbol{A}} + w_B\overline{\boldsymbol{B}}$. It is hence possible to use Eq. (4) to rewrite Eq. (3) in a more compact formulation assuming equal weights for each cycle:

$$\boldsymbol{R^t} = \frac{1}{N}\sum_{n=1}^{N}[\boldsymbol{R_n} + \rho(\overline{\boldsymbol{U}}_n - \overline{\boldsymbol{u}})(\overline{\boldsymbol{U}}_n - \overline{\boldsymbol{u}})^{\mathrm{T}}] = \frac{1}{N}\sum_{n=1}^{N}\boldsymbol{R_n} + \frac{\rho}{N}\sum_{n=1}^{N}\overline{\boldsymbol{U}}_n\,\overline{\boldsymbol{U}}_n^{\boldsymbol{T}} - \rho\overline{\boldsymbol{u}}\overline{\boldsymbol{u}}^{\boldsymbol{T}} \tag{5}$$

where $\boldsymbol{U_n} = (\boldsymbol{u_{n,1}}, \boldsymbol{u_{n,2}}, \dots, \boldsymbol{u_{n,m}}) \in \boldsymbol{R}^{3\times M}$ are the velocity measurements at a given cycle n and $\boldsymbol{R_n} = \rho\overline{\boldsymbol{U_n}'\boldsymbol{U_n}'}$ is the RST of the velocities at that given cycle. The values of $\boldsymbol{R_n}$ and $\overline{\boldsymbol{U}}_n$ can be directly stored while running the CFD simulation. For instantaneous measurements it was observed that 20 cardiac cycles were necessary to reach convergence in phase averaged quantities, however, only 10 cycles were necessary if time averaging $> 5ms$ was included. In this work, $\boldsymbol{R^t}$ is used as the CFD Reynolds stress tensor reference and various values for the averaging window ΔT were used to test the effect of time averaging on the generation of synthetic PC-MRI data.

The concept introduced with Eq. (4) can be extended to spatial downsampling operations of $\boldsymbol{R^t}$. Indeed, finding the value of the downsampled RST $\boldsymbol{R_\Delta^t}$ in a MRI voxel is equivalent to computing the RST of a union of disjoint velocity partitions with higher spatial resolutions within that voxel. By extending Eq. (5) for the case of spatial downsampling, $\boldsymbol{R_\Delta^t}$ becomes:

$$\boldsymbol{R_\Delta^t} = \boldsymbol{\mathcal{F}^{-1}}(\boldsymbol{\mathcal{F}}(\boldsymbol{R^t}) \circ \omega) + \rho\boldsymbol{\mathcal{F}^{-1}}(\boldsymbol{\mathcal{F}}(\overline{\boldsymbol{u}}\overline{\boldsymbol{u}}^{\boldsymbol{T}}) \circ \omega) - \rho\overline{\boldsymbol{u}}_{\boldsymbol{\Delta}}\overline{\boldsymbol{u}}_{\boldsymbol{\Delta}}^{\boldsymbol{T}} \tag{6}$$

where $\boldsymbol{\mathcal{F}}$ is the Fourier operator, $\circ$ is an apodization operator (defined as a truncated Gaussian window in this work) and $\overline{\boldsymbol{u}}_{\boldsymbol{\Delta}} = \boldsymbol{\mathcal{F}}^{-1}(\boldsymbol{\mathcal{F}}(\overline{\boldsymbol{u}}) \circ \omega)$ is the downsampled velocity. In Eq. (6), the three terms correspond to those in Eq. (5).

Details on Bayesian unfolding

A multipoint approach was used to probe the velocity field at $m = \{m \in \mathbb{Z}, 0 \leq m \leq 3 = M\}$ different encoding strengths $\boldsymbol{k}_{v,i}^{\boldsymbol{m}}$ for each encoding direction i, where $|\boldsymbol{k}_{v,i}^{\boldsymbol{m}}| = \pi/[\mathrm{VENC}]_i^m$. This results in a 19-point encoding approach (a reference shot with $[\mathrm{VENC}]_i^0 = 0\ [m/s]$ and six encoding directions with three different encoding strengths). Values of VENC used in this work are found in Table S1, the third VENC value was chosen larger than the maximum velocity magnitude observed at peak systole to allow phase unwrapping [7].

		Velocity Encoding [m/s]		
		$\mathbf{VENC_1}$	$\mathbf{VENC_2}$	$\mathbf{VENC_3}$
**Multipoint Synthetic PC-MRI**	**Idealized Steady**	0.1	0.3	1
	**Idealized Pulsatile**	0.5	1.5	3
	**Healthy Aorta**	0.4	0.67	2
	**50% AS Aorta**	0.5	1.5	4.5
	**75% AS Aorta**	0.5	1.5	4.5
	**90% AS Aorta**	0.5	1.5	9

Table S1: Encoding strengths used for the synthetic multipoint datasets. For all multipoint data, an additional encoding with $VENC_0 = 0\ [ms^{-1}]$ is used as reference.

Bayesian analysis was then used to combine data from all measurements m. Thereby a joint probability map for both scalar parameters $\{v, \sigma\} = \theta$ is obtained for each voxel and each encoding direction [8]. The resulting posterior probability for the ith direction given the model I and the measured image data $\boldsymbol{D_i} = S^*(\boldsymbol{k}_{v,i}^{\boldsymbol{m}})$ is [9]:

$$P_i(\theta_i|\boldsymbol{D_i}, I) \propto \frac{1}{\lambda}\left[1 - \frac{h_1^2 + h_2^2}{\sum_{m=1}^{M}\left(\Re\left(D_i^{\boldsymbol{m}}\right)^2 + \Im\left(D_i^{\boldsymbol{m}}\right)^2\right)}\right]^{1-M} \tag{7}$$

$$h_1 = \frac{1}{\sqrt{\lambda}}\sum_{m=1}^{M}\left[\left(\Re(D_i^{\boldsymbol{m}})\cos\left(v\left|\boldsymbol{k}_{v,i}^{\boldsymbol{m}}\right|\right) - \Im(D_i^{\boldsymbol{m}})\sin\left(v\left|\boldsymbol{k}_{v,i}^{\boldsymbol{m}}\right|\right)\right)e^{-\frac{\sigma^2\left|\boldsymbol{k}_{v,i}^{\boldsymbol{m}}\right|^2}{2}}\right] \tag{8}$$

$$h_2 = \frac{1}{\sqrt{\lambda}}\sum_{m=1}^{M}\left[\left(\Re(D_i^{\boldsymbol{m}})\sin\left(v\left|\boldsymbol{k}_{v,i}^{\boldsymbol{m}}\right|\right) + \Im(D_i^{\boldsymbol{m}})\cos\left(v\left|\boldsymbol{k}_{v,i}^{\boldsymbol{m}}\right|\right)\right)e^{-\frac{\sigma^2\left|\boldsymbol{k}_{v,i}^{\boldsymbol{m}}\right|^2}{2}}\right] \tag{9}$$

$$\lambda = \sum_{m=1}^{M} e^{-\sigma^2\left|\boldsymbol{k}_{v,i}^{\boldsymbol{m}}\right|^2} \tag{10}$$

where the term $h_1^2 + h_2^2$ is the mean square projection of the data onto the model and $\Re$ and $\Im$ represent real and imaginary components, respectively. The contributions of each measurement to the global probability are weighted according to its magnitude and resulting sensitivity to errors due to noise. Phase wraps are inherently accounted for as long as one of the velocity-encoded images is free of aliasing. In order to compute the most likely values of v and σ, the posterior probability has to be maximized (Fig. 1e):

$$\{\hat{\boldsymbol{v}}, \hat{\boldsymbol{\sigma}}\} = \operatorname{argmin}_{v,\sigma} - \log\big(P_i(\theta_i|\boldsymbol{D_i}, I)\big) \tag{11}$$

where $\hat{\boldsymbol{v}} \in \mathbb{R}^{6\times1}$ and $\hat{\boldsymbol{\sigma}} \in \mathbb{R}^{6\times1}$ are the predicted velocity and standard deviation vectors. This scalar multivariate minimization is performed with Scipy using the Nelder-Mead algorithm [10] for each voxel in the region of interest and each encoding direction i. The minimization is performed as a post-processing step after running the CFD simulation and typically requires 0.25 CPU hours per 100k voxels. Finally, each voxel's Bayesian prediction $\{\hat{\boldsymbol{v}}, \hat{\boldsymbol{\sigma}}\} \equiv \left\{\breve{\boldsymbol{v}}, \boldsymbol{\sigma}_{\boldsymbol{k}_v}\right\}$ to reconstruct the synthetic MRI Reynolds stress tensor $\boldsymbol{R}^*$ and Cartesian velocity $\boldsymbol{u}^*$.

Bibliography

1. Grady, L. Random Walks for Image Segmentation. *IEEE Trans. Pattern Anal. Mach. Intell.* **28**, 1768–1783 (2006).

2. Chan, T. & Vese, L. An Active Contour Model without Edges. in 141–151 (1999). doi:10.1007/3-540-48236-9_13.

3. Liu, X. *et al.* A numerical study on the flow of blood and the transport of LDL in the human aorta: The physiological significance of the helical flow in the aortic arch. *Am. J. Physiol. - Hear. Circ. Physiol.* **297**, 163–170 (2009).

4. Löhner, R. & Yang, C. Improved ALE mesh velocities for moving bodies. *Commun. Numer. Methods Eng.* **12**, 599–608 (1996).

5. Perona, P. & Malik, J. Scale-space and edge detection using anisotropic diffusion. *IEEE Trans. Pattern Anal. Mach. Intell.* **12**, 629–639 (1990).

6. Schubert, E. & Gertz, M. Numerically stable parallel computation of (co-)variance. in *Proceedings of the 30th International Conference on Scientific and Statistical Database Management* 1–12 (ACM, 2018). doi:10.1145/3221269.3223036.

7. Walheim, J., Dillinger, H., Gotschy, A. & Kozerke, S. 5D Flow Tensor MRI to Efficiently Map Reynolds Stresses of Aortic Blood Flow In-Vivo. *Sci. Rep.* **9**, 1–12 (2019).

8. Bretthorst, G. L. Bayesian analysis. I. Parameter estimation using quadrature NMR models. *J. Magn. Reson.* **88**, 533–551 (1990).

9. Binter, C., Knobloch, V., Manka, R., Sigfridsson, A. & Kozerke, S. Bayesian multipoint velocity encoding for concurrent flow and turbulence mapping. *Magn. Reson. Med.* **69**, 1337–1345 (2013).

10. Gao, F. & Han, L. Implementing the Nelder-Mead simplex algorithm with adaptive parameters. *Comput. Optim. Appl.* **51**, 259–277 (2012).
